# Supplementary material for: Effects of short birth interval on different forms of child mortality in Bangladesh: Application of propensity score matching technique with inverse probability of treatment weighting
Source: PLoS One. 2023 Apr 21;18(4):e0284776. doi: 10.1371/journal.pone.0284776 (PMC10121045; doi:10.1371/journal.pone.0284776)
Supplement: Attachment — Submitted filename: Response to reviewers.docx [file pone.0284776.s001.docx]

**Journal Requirements:**

When submitting your revision, we need you to address these additional requirements.

1. Please ensure that your manuscript meets PLOS ONE's style requirements, including those for file naming. The PLOS ONE style templates can be found at 
[https://journals.plos.org/plosone/s/file?id=wjVg/PLOSOne_formatting_sample_main_body.pdf](https://protect-au.mimecast.com/s/CZD2CgZ0ygfOyAmZf39tnN?domain=journals.plos.org) and 
[https://journals.plos.org/plosone/s/file?id=ba62/PLOSOne_formatting_sample_title_authors_affiliations.pdf](https://protect-au.mimecast.com/s/2gMhCjZ1BlfKmn3Qf1haTV?domain=journals.plos.org).

**Our response: We have checked all contents of this manuscript and ensured journal’s formatting style.**

2. We suggest you thoroughly copyedit your manuscript for language usage, spelling, and grammar. If you do not know anyone who can help you do this, you may wish to consider employing a professional scientific editing service.

Whilst you may use any professional scientific editing service of your choice, PLOS has partnered with both American Journal Experts (AJE) and Editage to provide discounted services to PLOS authors. Both organizations have experience helping authors meet PLOS guidelines and can provide language editing, translation, manuscript formatting, and figure formatting to ensure your manuscript meets our submission guidelines. To take advantage of our partnership with AJE, visit the AJE website ([http://learn.aje.com/plos/](https://protect-au.mimecast.com/s/5BXDCk81gmU8BOrzc8u363?domain=learn.aje.com)) for a 15% discount off AJE services. To take advantage of our partnership with Editage, visit the Editage website ([www.editage.com](https://protect-au.mimecast.com/s/okyOClx10nU0E2ANUV4Trq?domain=editage.com)) and enter referral code PLOSEDIT for a 15% discount off Editage services.  If the PLOS editorial team finds any language issues in text that either AJE or Editage has edited, the service provider will re-edit the text for free.

Upon resubmission, please provide the following:

The name of the colleague or the details of the professional service that edited your manuscript

A copy of your manuscript showing your changes by either highlighting them or using track changes (uploaded as a *supporting information* file)

A clean copy of the edited manuscript (uploaded as the new *manuscript* file.

**Our response:** We would like to inform you that as per your advice we have carefully edited the manuscript to improve readability, clarity, and quality of the contents.

3. We note that you have indicated that data from this study are available upon request. PLOS only allows data to be available upon request if there are legal or ethical restrictions on sharing data publicly. For information on unacceptable data access restrictions, please see [http://journals.plos.org/plosone/s/data-availability#loc-unacceptable-data-access-restrictions](https://protect-au.mimecast.com/s/k9rHCmO5koUBljANI33uHt?domain=journals.plos.org).

In your revised cover letter, please address the following prompts:

a) If there are ethical or legal restrictions on sharing a de-identified data set, please explain them in detail (e.g., data contain potentially identifying or sensitive patient information) and who has imposed them (e.g., an ethics committee). Please also provide contact information for a data access committee, ethics committee, or other institutional body to which data requests may be sent.

b) If there are no restrictions, please upload the minimal anonymized data set necessary to replicate your study findings as either Supporting Information files or to a stable, public repository and provide us with the relevant URLs, DOIs, or accession numbers. Please see [http://www.bmj.com/content/340/bmj.c181.long](https://protect-au.mimecast.com/s/yZetCnx1lpUAD7KNSvaL1Z?domain=bmj.com) for guidelines on how to de-identify and prepare clinical data for publication. For a list of acceptable repositories, please see [http://journals.plos.org/plosone/s/data-availability#loc-recommended-repositories](https://protect-au.mimecast.com/s/yRV2CoV1mqfjYrB3s5dv35?domain=journals.plos.org).

We will update your Data Availability statement on your behalf to reflect the information you provide.

4. Your ethics statement should only appear in the Methods section of your manuscript. If your ethics statement is written in any section besides the Methods, please move it to the Methods section and delete it from any other section. Please ensure that your ethics statement is included in your manuscript, as the ethics statement entered into the online submission form will not be published alongside your manuscript.

**Our response: We have moved ethical approval section from end of the manuscript to the method section.**

5. Please review your reference list to ensure that it is complete and correct. If you have cited papers that have been retracted, please include the rationale for doing so in the manuscript text, or remove these references and replace them with relevant current references. Any changes to the reference list should be mentioned in the rebuttal letter that accompanies your revised manuscript. If you need to cite a retracted article, indicate the article’s retracted status in the References list and also include a citation and full reference for the retraction notice.

**Our response: We have revised our reference list. They are correct and complete.**

**Reviewers' comments:**

**Reviewer #1:** The paper worth to publish due to the following criteria:

1-The research is original and its done for first time in Bangladesh

2- The sample size is big enough to show a strong and more accurate result for research at the level of the country

3- The size of control is equal or more than the sample size

4-The statistical requirements can be seen in the required level.

5- The english of manuscript is good and far from mistakes.

**Our response: Many thanks.**

Recommendation:

I prefer to write the result is percentage in addition to the writing of the result (number of samples) per 1000. It can be put between brackets.

**Our response: We already presented estimate per 1000 children, they are not number. We understand the reviewer misunderstood me because we were not clear enough in mentioning this. We have now edited the table heading and clearly mentioned it.**

**Reviewer #2:** Effects of short birth interval on different forms of child mortality in Bangladesh: application of propensity score matching technique with inverse probability of treatment weighting

Summary and general impression:

The paper made by Mohammad Zahidul Islam et al. team had great and fruitful efforts to find out how SBI affects neonatal, infant, and under-five mortality. The large sample size and the method of analyzing the data using a propensity score matching model; it was wonderful and scientifically sound. It gave a good insight into the importance of SBI and its impact on the sustainable development goals progress in Bangladesh and the world.

Special issues:

Minor Revision:

1. One of the most important reasons for the death of children under five years of age is the occurrence of infections, accidents and other causes that may not be related in one way or another to death at this age. Has any data been collected on the causes of death for these children, whether from the records or by asking the mothers? If this is not done, I think it should be placed in limitations section of the study as one of the confounders.

**Our response: We agree with the reviewer that infection is an important reasons of child mortality. However, the surveys we analysed do not have these data. As suggested by the reviewer, we have now mentioned it in the limitation section. This reads-**

Moreover, though DAG was used to select covariates to be considered in this study, however, many potential covariates including child food habits, causes of deaths including infection status, society level norms and culture and surrounding environments, are not available in the surveys. Therefore, we could not consider them in the DAG and in the analysis.

2. The researchers mentioned that they divided the sample according to their residence and mentioned the relative weight of them, but I did not see that in the results, as well as whether there was a difference between the rural and urban residents.

**Our response: We have edited relevant sentences to address this issue.**

3. Was there an influence of some other important factors such as their social, religious and cultural position on birth spacing?

**Our response: We agree with the reviewer that these mentioned factors are important. However, due to lack of data we did not adjust them. We mentioned this in the limitation section of the manuscript. Please see our response above.**
