# Supplementary material for: Effects of short birth interval on different forms of child mortality in Bangladesh: Application of propensity score matching technique with inverse probability of treatment weighting
Source: PLoS One. 2023 Apr 21;18(4):e0284776. doi: 10.1371/journal.pone.0284776 (PMC10121045; doi:10.1371/journal.pone.0284776)
Supplement: Attachment — Submitted filename: PONE-D-22-19640_R2_Revised Manuscript with Track changes.docx [file pone.0284776.s002.docx]

| **Comments (Reviewer 3):** | **Response** |
| --- | --- |
| The manuscript needs a final check for minor typos, grammar and syntax (marked in track changes below). |  |
| 1. The work presented meets the requirements for publication. There were some unclear statements such as in the inclusive criteria for women in the survey –ii) passed the most recent night before the survey at the selected households - what does this mean? |  |
| 2. Please include the WHO interval range to classify SBI in the discussion. Do you think this SBI range is more applicable for Bangladesh context? If yes/no, why? |  |
| 3. The reference list needs to be checked for missing DOI and the correct format. |  |

**Effects of short birth interval on different forms of child mortality in Bangladesh: application of propensity score matching technique with inverse probability of treatment weighting**

Mohammad Zahidul Islam^1,2^, Md. Mostafizur Rahman^2^, Md. Nuruzzaman Khan^1^

1. Department of Population Science, Jatiya Kabi Kazi Nazrul Islam University, Trishal, Mymensingh, Bangladesh
2. Department of Population Science and Human Resource Development, Rajshahi University, Bangladesh

**Short running title:** Short birth interval and child mortality in Bangladesh

**Corresponding author**

Dr. Md Nuruzzaman Khan

Assistant Professor

Department of Population Science

Jatiya Kabi Kazi Nazrul Islam University, Mymensingh, Bangladesh

Email: [mdnuruzzaman.khan@uon.edu.au](mailto:mdnuruzzaman.khan@uon.edu.au)

Mob: +08801737151554

**Abbreviations**

LMICs, low- and middle-income countries; SBI, short birth interval; aPR, adjusted prevalence ratio; MDGs, Millennium Development Goals; SDGs, Sustainable Development Goals; BDHS, Bangladesh Demographic and Health Survey; BHFS, Bangladesh Health Facility Survey; DHF, district health facilities; MCWC, mother and child welfare centre; DAG, Directed Acyclic Graph

**Abstract**

**Objectives:** Prevalence of Short Birth Interval (SBI) is higher in Low- and Middle-Income countries (LMICs), including Bangladesh. Previous studies in LMICs estimated the effects of SBI on child mortality by comparing two unequal groups of mothers according to their socio-economic status. This may overestimate or underestimate the true effect of birth interval on child mortality when the sample size is relatively small. We determined the effects of SBI on several forms of child mortality in Bangladesh by comparing two equal groups created by applying the propensity score matching technique.

**Methods:** Data from 5,941 mothers and 1594 health facilities were analysed in this study, extracted from the 2017/18 Bangladesh Demographic and Health Survey and the 2017 Bangladesh Health Facility Survey. The exposure variable was SBI (interval between two subsequent births <33 months: yes, no). The outcome variables were neonatal mortality (mortality within 28 days of births: yes, no), infant mortality (mortality within 1 year of birth: yes, no) and under-five mortality (mortality within five years of births: yes, no). Multilevel Poisson regression based on inverse probability treatment weights was used to determine the association between exposure and outcome variables.

**Results:** The prevalence rate of neonatal, infant and under-five mortality was 48.8, 30.8, and 23.1 per 1000 live births, respectively. Around 63% (aPR, 1.63; 95% CI, 1.08-2.46) higher likelihood of neonatal mortality was found among newborns of SBI mothers than newborns of non-SBI mothers. The prevalence of infant mortality and under-five mortality were 1.45 times (aPR,1.45; 95% CI, 1.01-2.08) and 2.82 times (aPR,2.82; 95% CI, 2.16-3.70) higher among babies born in a short interval of their immediately preceding sibling as compared to the babies born in a normal interval of their immediately preceding sibling.

**Conclusion:** The findings indicate that SBI is an important predictor of child mortality. Consequently, around 1 million children born in a short interval every year in Bangladesh are at risk of dying before reaching their fifth birthday. This indicates a challenge for Bangladesh to achieve the SDG 3 target to reduce neonatal and under-five mortality to 12 and 25 deaths per 1000 live births, respectively. Hence, awareness-building programs about the adverse effects of SBI and strengthening existing healthcare facilities are important.

**Keywords:** short birth interval, neonatal mortality, infant mortality, child mortality, inverse probability of treatment waiting, propensity score matching technique

# **Introduction**

The world has observed a remarkable reduction in under-five mortality during the Millennium Development Goals (MDGs) period of 2000 to 2015 [1, 2]. The global under-five mortality rate declined to 43 per 1000 live births in 2015, from 75 per 1000 live births in 2000 [2]. This was equivalent to around a 32% drop in 15 years, with 1 in 13 children dying before age 5 in 2000, compared to 1 in 23 in 2015 [3]. However, this progress was insufficient to achieve the MDG target of a two-thirds reduction of under-five mortality. By the end of the MDGs in 2015, around 5.9 million under-five deaths occurred worldwide, with an estimated 16,000 deaths every day [3]. The progress was insufficient in sub-Saharan Africa and South Asia particularly, where around 81% of the global under-five deaths occurred in 2015 [1, 3]. Around half of these deaths are found to occur within 28 days of life, with a 5% increase from 40% in 1990. In this circumstance, in 2015, the world leaders set a Sustainable Development Goals (SDGs) target to reduce neonatal and under-five mortality to 12 and 25 per 1000 live births respectively, by 2030 [4]. However, in 2022, , just 17 and 37 neonatal and under-five deaths were recorded per 1000 live births, respectively [5]. Importantly, the rate of reduction for the first half of the SDGs period, from 2015-2021, was not adequate to achieve the SDGs child mortality targets by 2030; and was even lower than the rates of reduction for the equal number of years in the MDGs’ period in respect to the achieving the relevant MDGs’ targets [5]. Therefore, the lack of progress in reducing child mortality rates is an ongoing public health threat, particularly in sub-Saharan Africa and South Asia, despite interventions at the maternal and health facilities that can help these regions to prevent almost all of these deaths [3].

Bangladesh, a low- and middle-income country (LMICs), is the second major contributor to the South Asian under-five deaths toll [6], with current estimated neonatal and under-five mortality rates at 17 (95% CI: 15-20) and 29 (95% CI: 26, 32) per 1000 live births, respectively [7]. Bangladesh was among the few countries that achieved the MDGs target for under-five mortality, and its high number of yearly births of around 4 million is second after Pakistan [8]. The population momentum has created a bulge in the younger generation in Bangladesh, as well as other South Asian countries and LMICs [9]. Therefore, it is expected that the present number of births will continue to grow. This indicates Bangladesh will continue contributing to higher numbers of under-five deaths and unless preventive action is taken, the country will not meet the SDGs targets by 2030 [10]. Bangladesh needs to strengthen its healthcare facilities to ensure effective maternal healthcare services use and address pregnancy-related complications and deaths [10]. Population-level challenges, including early marriage, immunization coverage, maternal and under-five children under-nutrition, violence against children and improvement of water and sanitation, must also be prioritised in existing policies and programs [11]. A similar recommendation can be made for other LMICs [11].

Short Birth Interval (SBI), defined as less than 33 months interval between two subsequent births, is found to be linked to multiple factors affecting under-five mortality, including early marriage, pregnancy complications, and maternal and under-five children nutritional disorders. This linkage may arise in direct and indirect ways: (i) women having these characteristics, i.e., early marriage, prefer births in short intervals, and (ii) short interval births which increase adverse outcomes, such as pregnancy complications, and maternal and under-five nutrition. The associations are mediated by other factors, including the disadvantaged socioeconomic conditions of the mothers, previous child survival status and the distance from home to nearby clinics [12-15]. These linkages indicate a potential association between SBI and under-five mortality. A systematic review and meta-analysis of 51 studies conducted in LMICs by this author also found a significant linkage of SBI with stillbirths, neonatal mortality, infant mortality and under-five mortality [16]. However, the review included articles based on secondary cross-sectional data with a relatively small sample size for mortality events and had at least two mentionable limitations. First, the estimated mortality was produced by comparing two unequal groups with respect to the socio-demographic factors, which are independent predictors of child mortality. The influence of health facility-level factors, also an important predictors of child mortality, was mostly ignored in the published articles, which is the second major limitation [14]. Besides, some other mentionable limitations of the published papers were the consideration of different intervals to define SBI, a small list of confounding factors and the analysis of a sample of rural or urban areas [17-20]. A recent study conducted in Ethiopia tried to address the limitation of comparing socio-demographically unequal groups; however, the authors of this paper did not address the health facility level factors [21]. The present study aimed to determine the effects of SBI on neonatal, infant and under-five mortality. The effects were estimated by analyzing nationally representative data so that mothers of similar socio-demographic factors were compared along with the adjustment of potential confounders.

# **Methods**

**Study design and sampling**

The 2017/18 Bangladesh Demographic and Health Survey (BDHS) and the 2017 Bangladesh Health Facility Survey (BHFS) data were linked and analysed in this study. Both are nationally representative surveys conducted as part of the Demographic and Health Survey Program of the USA. The details of these surveys have been published elsewhere [22, 23], with key aspects of these surveys presented here.

The 2017/18 BDHS was the seventh round of BDHS conducted by a government-owned Bangladeshi research firm, the National Institute of Population Research and Training, along with a private research firm, Mitra and Associates. The Demographic and Health Survey Program of the USA provided the overall supervision. The international development partners of Bangladesh, including the UNFPA and USAID, provided the financial and technical support to conduct this survey.

The reproductive-aged married women were the main sample in this survey, included from the nationally representative selected households. The conditions applied for women to be included in the survey were (i) usual residents of the selected household or (ii) passed the most recent night before the survey at the selected households. The households were selected in two stages. In the first stage, a total of 675 clusters were selected randomly from a list of 293,579 clusters of Bangladesh generated by the Bangladesh Bureau of Statistics as part of the 2011 Bangladesh National Population Census. Three clusters were excluded due to flood and the remaining 672 clusters were included in the survey. A fixed number of 30 households from each cluster was selected at the second stage of sampling. This produced a list of 20,160 households, of which the survey was conducted in 19,457 households with an over 96% inclusion rate. There were 20,376 eligible women in these selected households, and 20,127 women were interviewed with a response rate of 98.8%.

The 2017 BHFS used a list of 19,811 registered health facilities as a sampling frame generated by the Ministry of Health and Family Welfare. A total of 1,600 health facilities were selected from this list and 1,524 health facilities were finally included in the survey. The selection was made following the census of the district health facilities (DHF) and mother and child welfare centre (MCWC) and stratified random sampling of other healthcare facilities of the government, private, and non-governmental organizations [23].

**Study sample**

The BDHS recorded birth interval data for 5,941 women analysed in this study. The inclusion criteria for this sample were: (i) women who had at least two pregnancies, of which the most recent one ended with live birth within five years of the survey date, (ii) the second most recent pregnancy ended with live birth or termination, and (ii) the end dates of pregnancies and the interval before the most recent live birth were recorded.

**Outcome variable**

We considered three outcome variables: neonatal mortality (death occurred within 1 month of live birth), infant mortality (deaths occurred within one year of live birth) and under-five mortality (death occurred within five years of live births). The BDHS recorded these mortality data by asking women whether they had any live birth within five years of the survey date and the survival status of their respective children. In the occurrence of more than one live birth within five years, the survival status data were collected for every child. These data were then categorized using WHO guidelines to generate the outcome variables. The exclusion criteria was multiple pregnancies because of their different death patterns, and only singleton pregnancies were analysed [24, 25].

**Explanatory variables**

The primary explanatory variable was SBI, defined as an interval of <33 months between the two most recent births as recommended by the WHO [26]. The BDHS recorded this data in months by subtracting the date of birth of the most recent child from the date of birth or termination of the second most recent child. Relevant data were recorded during the survey by seeing birth registration reports or immunization card. If none were available, the mothers were requested to recall their memories by referring to memorable events like floods or the national election.

We conducted a comprehensive literature search in Medline, Embase, Web of Science, CINHAL, and Google Scholar to identify the key factors to study the association between birth interval and under-five mortality. For this, special attention was paid to the studies conducted in Bangladesh [17-20, 27, 28] and the studies conducted in some other LMICs [29-34]. The availability of the selected variables in the survey was then checked. Finally, the selected variables whose data were available in the survey were then included in the DAG (Directed Acyclic Graph, Figure 1), where interrelationships among the included variables were considered carefully by the expert researchers in this group (Khan MN and Rahman MM). This produced a list of confounding factors to be studied in the relationship, which we considered in our analysis. The factors were maternal age at birth, maternal education, maternal formal employment status, partner’s educational status, sex of the household’s head, total children ever born, and exposure to mass media. The sex of the deceased child’s sibling was also considered.

**Note:** CEB, children ever born, Nm, neonatal mortality, IM, infant mortality, and U5M, under-five mortality

**Figure 1: Direct acyclic graph used to select controlling variables to study the relationship of the short birth interval with neonatal, infant and under-five mortality.**

**Statistical analysis**

Descriptive statistics, frequency and mean, was used to describe the characteristics of the respondents. Considering the relatively small data for neonatal, infant and under 5 mortality (rare events), we applied the propensity score matching technique with inverse probability of treatment weighting to estimate the unbiased effect of short birth interval on each outcome [21]. This method is the best because compared to other propensity score methods, it preserves the sample size of the original data and provides an appropriate estimation of the variance of the main effect and maintains an appropriate type I error rate [35-37]. Given observed baseline covariates, the propensity score assigned the probability of treatment among the observations after adjusting the confounders due to non-random treatment [21]. Inverse probability of treatment weighting aided in reducing the observations using matching for the confounder adjustment and providing weights to the entire study sample depending on the conditional probability of treatment [35, 38-40]. Details of how the propensity score with inverse probability of treatment weighting works was given elsewhere [21].

Since the BDHS employed a complex two-stage, stratified, clustered random sampling procedure, we applied the sampling weights to adjust the non-proportional allocation of sample participants to different clusters [22]. We used the multilevel Poisson regression model to estimate the treatment effect (short birth interval) on outcomes. Final estimates of the outcome variables were incorporated with the product of sampling weight and the stabilized weight by the inverse probability of treatment weighting. The incorporation of both weights is required to estimate the unbiased treatment effects which can be generalized to the original survey population. Effects were expressed in adjusted prevalence ratio (aPR) with their 95% confidence interval. All the analyses were performed in the Stata version 15.10 (StataCorp Stata Statistical Software: Release V.15. College Station, Texas: StataCorp LP 2017).

**Ethics approval and consent to participate:** The survey analysed was approved by the institutional review board of ICF and the National Research Ethics Committee of the Bangladesh Medical Research Council. Informed consent was obtained from all participants. All necessary patient/participant consent has been obtained and the appropriate institutional forms have been archived. No separate ethical approval was required to conduct this study. We obtained permission to access this survey and conduct this research. All methods were performed in accordance with the relevant guidelines and regulations.

**Results**

**Background characteristics of the respondents**

The key information about the study participants, exposure and outcome variables are presented in Table 1. The mean age of the mothers was 26 years, and the mean weight was 52 kilograms. The mean age of the child analysed was nearly 31 months. Around 26% of the total mothers analysed had short interval births. The under-five, infant and neonatal mortality rates were 48.8, 30.8 and 23.1 per 1000 live births, respectively.

**Table 1: Key information about the study participants, exposure, and outcome variables. SD Standard deviation, CI confidence interval.**

| **Demographics of mothers** | Frequency (n=5946) | Weighted percentage (95% CI) |
| --- | --- | --- |
| **Mean age in years (mean ± SD)** | 5946 | 25.93 (±5.13) |
| Mean weight in kilograms (mean ± SD) | 5860 | 52.04 (±10.38) |
| Mean years of education (mean ± SD) | 5946 | 6.12 (±3.70) |
| **Demographics of under-five children** |  |  |
| Mean age in months (mean ± SD) | 5946 | 30.55 (±17.77) |
| Girls | 5946 | 47.75 (46.40-49.11) |
| **Birth interval** |  |  |
| Birth in short interval | 1566 | 26.36 (24.84-27.94) |
| Birth not in short interval | 4375 | 73.64 (72.06-75.16) |
| **Outcomes** |  |  |
| Neonatal mortality per 1000 live births | 137 | 23.1 (19.1-28.0) |
| Infant mortality per 1000 live births | 182 | 30.8 (26.1-36.2) |
| Under-five mortality per 1000 live births | 289 | 48.8 (42.7-55.7) |

**Distribution of neonatal mortality, infant mortality, and under-five mortality by background characteristics**

Different distributions of the under-five, infant, and neonatal mortality are presented in Table 2. We found a higher prevalence of under-five mortality among comparatively less aged and illiterate mothers. The under-five mortality rate was also higher among mothers whose husbands had no education at the time of the survey, mothers who resided in male-headed households and mothers who had >2 children ever born. A similar distribution was reported for infant and neonatal mortality.

**Table 2: Neonatal, infant and under-five mortality rates by background characteristics, BDHS 2017/18**

| **Characteristics** | **Neonatal mortality rate per 1000 (95% CI)** | **Infant mortality rate per 1000 (95% CI)** | **Under-5 mortality rate per 1000 (95% CI)** |
| --- | --- | --- | --- |
| **Maternal age at birth** |  |  |  |
| ≤19 | 35.4 (19.3-64.1) | 46.0 (27.5-76.0) | 135.8 (106.0-172.4) |
| 20-34 | 21.9 (17.7-27.1) | 29.6 (24.6-35.6) | 42.4 (36.3-49.5) |
| ≥35 | 24.1 (11.7-49.2) | 27.3 (13.8-53.1) | 28.3 (14.7-53.9) |
| **Mother’s educational status** |  |  |  |
| No education | 28.0 (16.8-46.1) | 35.3 (22.1-56.0) | 58.7 (39.6-86.1) |
| Primary | 26.9 (19.2-37.5) | 37.4 (28.4-49.2) | 52.4 (42.0-65.2) |
| Secondary | 19.6 (14.5-26.4) | 25.9 (20.0-33.6) | 46.3 (38.2-56.1) |
| Higher | 22.6 (11.7-43.2) | 27.2 (15.2-48.4) | 39.4 (25.1-61.2) |
| **Mother’s working status** |  |  |  |
| Yes | 29.2 (22.8-37.2) | 35.0 (28.1-43.5) | 50.1 (41.6-60.1) |
| No | 17.8 (12.9-24.5) | 27.0 (21.1-34.6) | 47.7 (39.7-57.1) |
| **Partner’s educational status** |  |  |  |
| No education | 27.9 (17.8-43.5) | 38.7 (26.9-55.4) | 56.9 (42.8-75.3) |
| Primary | 23.0 (16.7-31.7) | 30.3 (22.8-40.3) | 45.9 (36.4-57.7) |
| Secondary | 19.9 (13.4-29.4) | 29.3 (21.0-40.7) | 51.8 (40.6-65.8) |
| Higher | 23.6 (14.5-38.2) | 25.2 (15.9-39.8) | 40.2 (28.3-56.7) |
| **Sex of the household’s head** |  |  |  |
| Male | 22.3 (18.0-27.6) | 30.8 (25.8-36.7) | 50.1 (43.6-57.4) |
| Female | 27.9 (17.5-44.2) | 30.7 (19.3-48.3) | 40.9 (27.6-60.0) |
| **Total children ever born** |  |  |  |
| ≤2 | 12.6 (0.9-1.8) | 16.7 (12.2-22.7) | 45.1 (37.0-54.9) |
| >2 | 34.4 (27.3-43.2) | 45.9 (37.7-55.7) | 52.7 (43.7-63.5) |
| **Exposure to mass media** |  |  |  |
| Little exposed | 20.9 (15.5-28.1) | 29.5 (23.1-37.5) | 49.2 (40.2-60.2) |
| Moderate exposed | 26.8 (20.5-35.0) | 33.3 (26.1-42.2) | 49.0 (40.1-59.7) |
| Highly exposed | 15.9 (0.9-29.6) | 25.4 (16.0-40.2) | 46.7 (33.0-65.8) |
| **Sex of the deceased children sibling** |  |  |  |
| Male | 20.9 (15.7-27.7) | 28.4 (22.3-36.3) | 50.9 (42.0-61.6) |
| Female | 25.2 (19.7-32.2) | 32.9 (26.8-40.4) | 46.8 (39.6-55.2) |

**Balance diagnosis**

**Propensity Score balance**

The balance of propensity score (PS) before and after weighting across treatment (navy blue line) and control group (red line) are presented in Figure 2. The figure shows an adequate balance of the PS distribution between the treatment group after weighting.

|  |  |
| --- | --- |
| Propensity score BEFORE weighting | Propensity score AFTER weighting |

**Figure 2: Balance of propensity scores (PS) before and after weighting across treatment and comparison groups.**

**Covariate balance**

The balance of the PS for the covariates included in this study before and after weighting across treatment and control groups are presented in Table 3. After weighting adjustment, standardized differences of all covariates were <1%. This indicates good balance and comparability between the women with and without SBI.

**Table 3: Covariates PS balance before and after weighting**

| **Covariates** | **Mean in treated group** | **Mean in comparison group** | **Standardized differences** |
| --- | --- | --- | --- |
| Maternal age at birth | 0.78 | 0.77 | 0.012 |
| Mother’s educational status | 1.57 | 1.59 | -0.027 |
| Mother’s working status | 0.46 | 0.47 | -0.013 |
| Partner’s educational status | 1.39 | 1.43 | -0.033 |
| Sex of the household’s head | 1.12 | 1.12 | -0.015 |
| Total children ever born | 1.51 | 1.48 | 0.019 |
| Exposure to mass media | 0.73 | 0.73 | -0.009 |
| Sex of the deceased child’s sibling | 1.11 | 1.10 | 0.009 |

**Effects of short birth interval on neonatal, infant and under-five mortality**

After ensuring the balance of covariates, the association of SBI with neonatal, infant and under-five mortality were estimated separately using a multi-level Poisson regression model in the weighted sample adjusted for covariates. Results are presented in Table 4. The adjusted estimated odd ratio of neonatal mortality was 63% higher (aPR,1.63; 95% CI, 1.08-2.46) among babies of the mothers who experienced SBI than the babies of the mothers who did not experience SBI. The likelihood of infant mortality was also found 45% (aPR,1.45; 95% CI, 1.01-2.08) higher among babies who were born in short intervals than their counterparts born in normal intervals. The reported effect was even strongest for under-five mortality. We found 2.82 times (aPR,2.82; 95% CI, 2.16-3.70) higher likelihood of under-five mortality among children of the mothers who had their births in short intervals as compared to their counterparts who had normal intervals of successive births.

**Table 4: The effect of short birth interval on neonatal, infant and under-five mortality in Bangladesh, BDHS 2017/18**

| **Treatment variable** | **Neonatal mortality** | | **aPR^+^ (95% CI)** |
| --- | --- | --- | --- |
| **Short birth interval** | No (%) | Yes (%) |  |
| No | 4283 (73.80) | 92 (66.71) | Ref |
| Yes | 1520 (26.20) | 46 (33.29) | 1.63 (1.08-2.46)^**^ |
| **Short birth interval** | **Infant mortality** | |  |
|  | No (%) | Yes (%) |  |
| No | 4250 (73.80) | 125 (68.48) | Ref |
| Yes | 1509 (26.20) | 58 (31.52) | 1.45 (1.01-2.08)^**^ |
| **Short birth interval** | **Under-five mortality** | |  |
|  | No (%) | Yes (%) |  |
| No | 4222 (74.87) | 150 (51.87) | Ref |
| Yes | 1417 (25.13) | 139 (48.13) | 2.82 (2.16-3.70)*** |

**Note:** ^+^Models adjusted with maternal age at birth, maternal education, maternal formal employment status, partner’s educational status, sex of the household’s head, total children ever born, exposure to mass media and sex of the deceased child’s sibling.

^**^p<0.05, ^***^p<0.01

**Discussion**

We aimed to estimate the effects of SBI on neonatal, infant and under-five mortality by analyzing nationally representative data in such a way that mothers of similar socio-demographic factors were compared along with the adjustment of potential covariates. We applied the propensity score matching technique with inverse probability of treatment weighting after specifying the set of explanatory variables using the DAGs. The associations were determined using the multi-level Poisson regression model. We found a higher likelihood of neonatal, infant, and under-five child mortality among the mothers who had a short birth interval compared to their counterparts with normal birth intervals. The reported associations were robust in several areas, including the use of advanced statistical modelling with an appropriate weighting and confounders selected by the DAG, and consideration of household survey data together with the health facility survey data . As far as we know, this study is the first of its kind in Bangladesh as well as in LMICs. We suggest that the SBI is a strong predictor of neonatal, infant and under-five mortality in Bangladesh.

The association reported in this study was consistent with the previous studies in LMICs, including Nigeria [29, 41, 42], Ethiopia [21, 31, 33, 43, 44], Ghana [45], India [27], and Pakistan [34]. However, our estimated prevalence rates were lower than previous studies. One reason could be our use of WHO interval to classify SBI in this study rather than the intervals used by previous authors, ranging from <18 to 24 months. The methodological advantage is also an important contribution to such variation. Considering the significance of the findings, the reported associations in this study should be given priority in relevant policy reforms in Bangladesh.

The association between the SBI and neonatal mortality can arise in several pathways. There is evidence that when mothers become pregnant in shorter intervals, they face several adverse outcomes during pregnancy and delivery. For instance, it was suggested that the occurrence of births in short intervals increases folate depletion/anomalies, maternal nutritional deficiencies [21, 46-48], uterine rupture, postpartum hemorrhage [49, 50], congenital anomalies [48, 50] and gestational diabetes [46, 51-53]. It is also found that the SBI increases the occurrence of cesarean delivery [53-56]. These adverse maternal health outcomes could increase the occurrence of adverse birth outcomes, such as small for gestational age [50], massive genital bleeding [57], birth asphyxia and hypoxia [53, 55], preterm birth, low birth weight, and neonatal sepsis [46, 51-53]. All adverse outcomes could lead to neonatal mortality, mostly soon after delivery- the period when over 70% of the total neonatal deaths and most under-five deaths occurred in LMICs and Bangladesh [3, 11].

Even if the child passes this critical stage, along with the demand for extra care due to these adverse consequences during the neonatal period, they face additional challenges following their presence into infancy. For instance, the birth of two children in a closer period increases competition among siblings for care and food. This is an important direction considering the prevalence of short birth intervals is high among mothers of lower socio-demographic conditions because of their, (i) lack of awareness about the importance of longer birth intervals, (ii) inadequate knowledge of family planning services and (iii) lower use of contraception, or more likely some combinations of three. Importantly, mothers of lower socio-demographic conditions also have limited capacity to buy nutritious foods for their multiple children; which could lead to a higher prevalence of nutritional deficiencyamong the children. Furthermore, these limitations create demand from the children for additional food, which could reduce the mothers’ capacity for infant feeding, either through the supply of formula milk or breastfeeding, the latter an important determinant of child survival [58]. However, even if the mothers manage to fulfill all of these, the duration and intensity of breastfeeding is always lower for children born in short intervals than children born in normal intervals. All these could affect infants’ nutritional status and resistance to infection and may lead them to death [58-64].

These adverse consequences continue along with the children’s increasing age, unless protective measures, such as breastfeeding, nutritional foods, and immunization, have been taken. However, since short interval births mainly occur among socio-economically disadvantaged mothers, it is unlikely that the mothers would take preventive measures except if they were counselled properly to do so. This needs educating mothers about the importance of childcare, particularly for the children born in shorter intervals. Healthcare facilities also need to play a major role in ensuring child healthcare services when needed and other preventive measures in a timely manner, such as immunization. However, there is evidence that children born in short intervals are less likely to receive immunization with continuity [31, 32, 44, 65, 66], mostly because of their mothers’ lack of time and motivation to bring their children to the health centre [21]. This increases the occurrence of infectious diseases, including diarrhea and pneumonia. These, along with the existing adverse consequences, can increase under-five mortality.

Our findings imply that there needs to be substantial investment in health promotion to raise awareness about the impotence of longer interval births and adverse outcomes associated with short interval births. The healthcare delivery system also needs to be strengthened to ensure child healthcare services. However, these are challenging for Bangladesh, like other LMICs, for several reasons. The major challenge comes from the facility level, where protective measures, such as providing treatment, are prioritized over preventive measures, such as awareness building. In the current scenario of Bangladesh, mass media and family planning workers are playing an important role in awareing people about the adverse health events, including the importance of longer birth interval, however, the effectiveness of both ways are declining rapidly [67, 68]. The underlying reasons are mismatches between program timing and respondents’ availability, unattractive program content and people’s interest in other mass media where healthcare messages do not provide adequately [68]. The focus on field-level family planning services and monitoring family planning activities at the field level have also declined over the years [9]. Consequently, respondents’ options to get healthcare knowledge and contraception accessibility, which is an important determinant of short birth intervals, have been narrowed over the years [9]. This influences the socio-demographic disadvantaged women mostly, among which births in short interval are highly prevalent [14, 15, 69, 70].

The current study has several strengths and a few limitations. First, the methodological approach used in this study, propensity score matching with inverse probability weighting, helped us generate the estimate by comparing two equal groups, like a randomized control trial. Second, we analyzed data from two nationally representative households and health facility survey with adequate sample sizes. Third, the covariates were selected using DAGs, where all possible linkages among the covariates are considered. These helped us to generate robust estimates. However, due to the cross-sectional nature of the study, the findings reported could be temporary. Another limitation of this study is that though propensity score-based analyses were used, due to non-randomized study design, it may not account for unknown confounders in the same way that a randomized trial does. So the effects of residual confounders may not be avoided. Moreover, though DAG was used to select covariates in this study, many other covariates, including children food habits, causes of deaths including infection status, society level norms and culture and surrounding environments, were not available from the surveys.. However, despite the limitations, this study has novelty, and its findings could help the policy directions in Bangladesh and other LMICs to improve its child, maternal and reproductive health programs.

**Conclusion**

SBI was found as the strongest predictors of neonatal, infant and under-five mortality following the adjustment of potential confounders. This indicates that one in every four children in Bangladesh with a short birth interval, are at risk of mortality before reaching their fifth birthday. The SDGs targeted a significant reduction of neonatal and under-five mortality by 2030. With the current high prevalence of SBI in Bangladesh and subsequent mortality risks, the country is at risk of missing the targets unless the prevalence of SBI is addressed by ensuring family planning services and contraception methods are made accessible. Healthcare facility also needs to be strengthened to ensure an adequate preventive and curative cure for mothers and children. Awareness-building programs at the community level about the adverse effects of SBI should also be given priority.

**Acknowledgements:** We acknowledge the DHS of the USA for approving us to use their data.

**Funding:** None

**Authors’ contributions:** Islam MZI and Khan MN designed the study, performed the data analysis, and wrote the first draft of this manuscript. Khan MN and Rahman MM critically reviewed and edited all versions of this manuscript. All authors approved this final version of the manuscript.

**Competing of interest:** None.

**Consent for publication:** Informed consent was obtained from all participants during the survey using a standard DHS consent form.

**Availability of data and materials:** The data that support the findings of this study are available from The DHS Program and legal restrictions apply to the availability of these data, which were used under license for the current study, and so are not publicly available. Data are, however, available from The DHS Program (https://dhsprogram.com) upon submission of research proposal and/or reasonable request. To access the relevant data used in this study, interested authors need to submit research proposal based on the 2017/18 BDHS and 2017 BHFS. The variables used in this study are directly available in the datasets, so interested authors can easily find out the variables for analysis. No special privileges is required for this and the authors of this study did not have this.

# **References**

1.	Child mortality (under 5 years) [Internet]. The World Health Organization 2020 [cited July 11, 2022].

2.	You D, Hug L, Ejdemyr S, Idele P, Hogan D, Mathers C, et al. Global, regional, and national levels and trends in under-5 mortality between 1990 and 2015, with scenario-based projections to 2030: a systematic analysis by the UN Inter-agency Group for Child Mortality Estimation. The Lancet. 2015;386(10010):2275-86.

3.	In 2015, the global child mortality rate is less than half its 1990 levels, but the MDG 4 target has not been met [Internet]. 2015 (URL?)

4.	Sachs JD. From millennium development goals to sustainable development goals. The Lancet. 2012;379(9832):2206-11.

5.	Hug L, Alexander M, You D, Alkema L, for Child UI-aG. National, regional, and global levels and trends in neonatal mortality between 1990 and 2017, with scenario-based projections to 2030: a systematic analysis. The Lancet Global Health. 2019;7(6):e710-e20.

6.	Nahian Rahman M, Kabir R, Halima O, Alam MR, Al Mamun MA, Islam H. Under five child mortality & its risk factors in Bangladesh and other South Asian countries: a literature review. Ind J Publi c Health Res Dev. 2020;11(6):1374-9.

7.	United Nations, Inter-agency Group for Child Mortality Estimation. Levels & Trends in Child Mortality: Report 2021, Estimates developed by the United Nations Inter-agency Group for Child Mortality Estimation. New York: United Nations Children’s Fund, 2021.

8.	New Year’s Babies: Over 97,000 children will be born in South Asia on New Year’s Day [Internet]. 2022. (URL)

9.	Khan MN, Islam MM. Exploring rise of pregnancy in Bangladesh resulting from contraceptive failure. Scientific Reports. 2022;12(1):1-10.

10.	Khan MN, Trisha NI, Rashid MM. Availability and readiness of healthcare facilities and their effects on under-five mortality in Bangladesh: Analysis of linked data. Journal of Global Health, 2022:04082.

11.	Children in South Asia [Internet]. 2022 (URL)

12.	Tuz-Zahura F, Sen KK, Nilima S, Bari W. Can women's 3E index impede short birth interval? evidence from Bangladesh Demographic and Health Survey, 2017-18. PLoS One. 2022;17(1):e0263003.

13.	Nkonki L, Islam MZ, Islam MM, Rahman MM, Khan MN. Prevalence and risk factors of short birth interval in Bangladesh: Evidence from the linked data of population and health facility survey. PLOS Global Public Health. 2022;2(4):e0000288.

14.	Islam MZ, Islam MM, Rahman MM, Khan MN. Prevalence and risk factors of short birth interval in Bangladesh: Evidence from the linked data of population and health facility survey. PLOS Global Public Health. 2022;2(4):e0000288.

15.	Islam MZ, Islam MM, Rahman M, Khan M. Exploring hot spots of short birth intervals and associated factors using a nationally representative survey in Bangladesh. Scientific Reports. 2022;12(1):1-10.

16.	Islam MZ BM, Islam MM, Rahman MM, Khan MN. Negative effects of short birth interval and child mortality in low- and middle-income countries: a systematic review and meta-analysis 2022. (doi?)

17.	de Jonge HC, Azad K, Seward N, Kuddus A, Shaha S, Beard J, et al. Determinants and consequences of short birth interval in rural Bangladesh: a cross-sectional study. BMC Pregnancy Childbirth. 2014;14(1):427.

18.	Alam N. Birth spacing and infant and early childhood mortality in a high fertility area of Bangladesh: age-dependent and interactive effects. J Biosoc Sci. 1995;27(4):393-404.

19.	Miller JE, Trussell J, Pebley AR, Vaughan B. Birth spacing and child mortality in Bangladesh and the Philippines. Demography. 1992;29(2):305-18.

20.	Koenig MA, Phillips JF, Campbell OM, D'Souza S. Birth intervals and childhood mortality in rural Bangladesh. Demography. 1990;27(2):251-65.

21.	Shifti DM, Chojenta C, Holliday E, Loxton D. Effects of short birth interval on neonatal, infant and under-five child mortality in Ethiopia: a nationally representative observational study using inverse probability of treatment weighting. BMJ Open. 2021;11(8):e047892.

22.	National Institute of Population Research and Training (NIPORT) MaA, and ICF International. Bangladesh Demographic and Health Survey 2017/18. Dhaka, Bangladesh, and Rockville, Maryland, USA: NIPORT, Mitra and Associates, and ICF International. 2021.

23.	National Institute of Population Research and Training (NIPORT) MaA, and ICF International. Bangladesh Health Facility Survey 2017. Dhaka, Bangladesh: NIPORT, ACPR, and ICF. 2019.

24.	Kalikkot Thekkeveedu R, Dankhara N, Desai J, Klar AL, Patel J. Outcomes of multiple gestation births compared to singleton: analysis of multicenter KID database. Maternal health, neonatology and perinatology. 2021;7(1):1-11.

25.	Santana DS, Silveira C, Costa ML, Souza RT, Surita FG, Souza JP, et al. Perinatal outcomes in twin pregnancies complicated by maternal morbidity: evidence from the WHO Multicountry Survey on Maternal and Newborn Health. BMC Pregnancy and Childbirth. 2018;18(1):1-11.

26.	World Health Organization. Report of a WHO technical consultation on birth spacing. Geneva: World Health Organization, 2007.

27.	Houweling TAJ, van Klaveren D, Das S, Azad K, Tripathy P, Manandhar D, et al. A prediction model for neonatal mortality in low- and middle-income countries: an analysis of data from population surveillance sites in India, Nepal and Bangladesh. Int J Epidemiol. 2019;48(1):186-98.

28.	Akter S, Rahman JA, Rahman MM, Abedin S. The influence of birth spacing on child survival in Bangladesh: a life table approach. World Health Popul. 2010;12(1):42-56.

29.	Akinyemi JO, Bamgboye EA, Ayeni O. Trends in neonatal mortality in Nigeria and effects of bio-demographic and maternal characteristics. BMC Pediatr. 2015;15(1):36.

30.	Biradar R, Patel KK, Prasad JB. Effect of birth interval and wealth on under-5 child mortality in Nigeria. Clinical Epidemiology and Global Health. 2019;7(2):234-8.

31.	Fenta SM, Fenta HM, Ayenew GM. The best statistical model to estimate predictors of under-five mortality in Ethiopia. Journal of Big Data. 2020;7(1).

32.	Gebremichael SG, Fenta SM. Under-Five Mortality and Associated Risk Factors in Rural Settings of Ethiopia: Evidences from 2016 Ethiopian Demographic and Health Survey. Advances in Public Health. 2020;2020:1-13.

33.	Tesema GA, Worku MG, Teshale AB. Duration of birth interval and its predictors among reproductive-age women in Ethiopia: Gompertz gamma shared frailty modeling. PLoS One. 2021;16(2):e0247091..

34.	Afshan K, Narjis G, Qureshi IZ, Cappello M. Social determinants and causes of child mortality in Pakistan: Analysis of national demographic health surveys from 1990 to 2013. J Paediatr Child Health. 2020;56(3):457-72.

35.	Austin PC, Mamdani MM. A comparison of propensity score methods: a case-study estimating the effectiveness of post-AMI statin use. Stat Med. 2006;25(12):2084-106.

36.	Lee Y, Hong I, Lee MJ, Park HY. Identifying Risk of Depressive Symptoms in Adults With Physical Disabilities Receiving Rehabilitation Services: Propensity Score Approaches. Ann Rehabil Med. 2019;43(3):250-61.

37.	Xu S, Ross C, Raebel MA, Shetterly S, Blanchette C, Smith D. Use of stabilized inverse propensity scores as weights to directly estimate relative risk and its confidence intervals. Value Health. 2010;13(2):273-7.

38.	Austin PC. A Tutorial and Case Study in Propensity Score Analysis: An Application to Estimating the Effect of In-Hospital Smoking Cessation Counseling on Mortality. Multivariate Behav Res. 2011;46(1):119-51..

39.	Deb S, Austin PC, Tu JV, Ko DT, Mazer CD, Kiss A, et al. A Review of Propensity-Score Methods and Their Use in Cardiovascular Research. Can J Cardiol. 2016;32(2):259-65.

40.	Guo S, Fraser MW. Propensity score analysis: Statistical methods and applications: SAGE publications; 2014.

41.	Patel KK, Prasad JB, Biradar RA. Trends in and determinants of neonatal and infant mortality in Nigeria based on Demographic and Health Survey data. J Biosoc Sci. 2021;53(6):924-34.

42.	Morakinyo OM, Fagbamigbe AF. Neonatal, infant and under-five mortalities in Nigeria: An examination of trends and drivers (2003-2013). PLoS One. 2017;12(8):e0182990. Epub 2017/08/10.

43.	Aychiluhm SB, Tadesse AW, Mare KU, Abdu M, Ketema A. A multilevel analysis of short birth interval and its determinants among reproductive age women in developing regions of Ethiopia. PLoS One. 2020;15(8):e0237602.

44.	Tesema GA, Tessema ZT, Tamirat KS, Teshale AB. Complete basic childhood vaccination and associated factors among children aged 12-23 months in East Africa: a multilevel analysis of recent demographic and health surveys. BMC Public Health. 2020;20(1):1837.

45.	Kayode GA, Ansah E, Agyepong IA, Amoakoh-Coleman M, Grobbee DE, Klipstein-Grobusch K. Individual and community determinants of neonatal mortality in Ghana: a multilevel analysis. BMC Pregnancy Childbirth. 2014;14(1):165.

46.	Conde-Agudelo A, Rosas-Bermudez A, Castano F, Norton MH. Effects of birth spacing on maternal, perinatal, infant, and child health: a systematic review of causal mechanisms. Stud Fam Plann. 2012;43(2):93-114.

47.	Conde-Agudelo A, Rosas-Bermudez A, Kafury-Goeta AC. Effects of birth spacing on maternal health: a systematic review. Am J Obstet Gynecol. 2007;196(4):297-308.

48.	Chen I, Jhangri GS, Chandra S. Relationship between interpregnancy interval and congenital anomalies. Am J Obstet Gynecol. 2014;210(6):564 e1-8.

49.	Rao J, Fan D, Ma H, Lin D, Zhang H, Zhou Z, et al. Is there an optimal inter-delivery interval in women who underwent trial of labor after cesarean delivery (TOLAC)? Reprod Health. 2022;19(1):14.

50.	Ekin A, Gezer C, Taner CE, Ozeren M, Mat E, Solmaz U. Impact of interpregnancy interval on the subsequent risk of adverse perinatal outcomes. J Obstet Gynaecol Res. 2015;41(11):1744-51.

51.	Ahrens KA, Nelson H, Stidd RL, Moskosky S, Hutcheon JA. Short interpregnancy intervals and adverse perinatal outcomes in high-resource settings: An updated systematic review. Paediatr Perinat Epidemiol. 2019;33(1):O25-O47.

52.	Kangatharan C, Labram S, Bhattacharya S. Interpregnancy interval following miscarriage and adverse pregnancy outcomes: systematic review and meta-analysis. Hum Reprod Update. 2017;23(2):221-31.

53.	Shachar BZ, Lyell DJ. Interpregnancy interval and obstetrical complications. Obstet Gynecol Surv. 2012;67(9):584-96.

54.	Hutcheon JA, Nelson HD, Stidd R, Moskosky S, Ahrens KA. Short interpregnancy intervals and adverse maternal outcomes in high-resource settings: An updated systematic review. Paediatr Perinat Epidemiol. 2019;33(1):O48-O59.

55.	Ye L, Cao W, Yao J, Peng G, Zhou R. Systematic review of the effects of birth spacing after cesarean delivery on maternal and perinatal outcomes. Int J Gynaecol Obstet. 2019;147(1):19-28.

56.	O'Neill SM, Kearney PM, Kenny LC, Henriksen TB, Lutomski JE, Greene RA, et al. Caesarean delivery and subsequent pregnancy interval: a systematic review and meta-analysis. BMC Pregnancy Childbirth. 2013; 13:165.

57.	Ryu N, Ogawa M, Matsui H, Usui H, Shozu M. The clinical characteristics and early detection of postpartum choriocarcinoma. Int J Gynecol Cancer. 2015; 25(5):926-30.

58.	Lassi ZS, Rind F, Irfan O, Hadi R, Das JK, Bhutta ZA. Impact of Infant and Young Child Feeding (IYCF) Nutrition Interventions on Breastfeeding Practices, Growth and Mortality in Low- and Middle-Income Countries: Systematic Review. Nutrients. 2020;12(3):722.

59.	Dewey KG, Cohen RJ. Does birth spacing affect maternal or child nutritional status? A systematic literature review. Matern Child Nutr. 2007;3(3):151-73.

60.	Stuebe A. The risks of not breastfeeding for mothers and infants. Rev Obstet Gynecol. 2009;2(4):222-31.

61.	Victora CG, Bahl R, Barros AJ, França GV, Horton S, Krasevec J, et al. Breastfeeding in the 21st century: epidemiology, mechanisms, and lifelong effect. Lancet. 2016;387(10017):475-90.

62.	Boerma JT, Bicego GT. Preceding birth intervals and child survival: searching for pathways of influence. Stud Fam Plann. 1992;23(4):243-56. Epub 1992/07/01.

63.	Boerma T, Requejo J, Victora CG, Amouzou A, George A, Agyepong I, et al. Countdown to 2030: tracking progress towards universal coverage for reproductive, maternal, newborn, and child health. The Lancet. 2018;391(10129):1538-48.

64.	Victora CG, Requejo JH, Barros AJ, Berman P, Bhutta Z, Boerma T, et al. Countdown to 2015: a decade of tracking progress for maternal, newborn, and child survival. Lancet. 2016;387(10032):2049-59.

65.	Chowdhury AH, Hanifi SMA, Bhuiya A. Social determinants of under-five mortality in urban Bangladesh. Journal of Population Research. 2020;37(2):161-79.

66.	Rahman M, Obaida-Nasrin S. Factors affecting acceptance of complete immunization coverage of children under five years in rural Bangladesh. Salud Publica Mex. 2010;52(2):134-40. Epub 2010/05/21.

67.	Grilli R, Ramsay C, Minozzi S. Mass media interventions: effects on health services utilisation. Cochrane database of systematic reviews. 2002;(1).

68.	Alam MZ, Hu W, Uddin A. Digital transformation in healthcare services sector of bangladesh: current status, challenges and future direction. Journal on Innovation and Sustainability RISUS. 2020;11(1):30-8.

69.	De Jonge HC, Azad K, Seward N, Kuddus A, Shaha S, Beard J, et al. Determinants and consequences of short birth interval in rural Bangladesh: a cross-sectional study. BMC pregnancy and childbirth. 2014;14(1):1-7.

70.	Chirwa TF, Mantempa JN, Kinziunga FL, Kandala JD, Kandala N-B. An exploratory spatial analysis of geographical inequalities of birth intervals among young women in the Democratic Republic of Congo (DRC): a cross-sectional study. BMC pregnancy and childbirth. 2014;14(1):1-10.
