# Supplementary material for: Effects of short birth interval on different forms of child mortality in Bangladesh: Application of propensity score matching technique with inverse probability of treatment weighting
Source: PLoS One. 2023 Apr 21;18(4):e0284776. doi: 10.1371/journal.pone.0284776 (PMC10121045; doi:10.1371/journal.pone.0284776)
Supplement: Attachment — Submitted filename: Effects of short birth interval on different forms of child mortality in Bangladesh.docx [file pone.0284776.s003.docx]

Effects of short birth interval on different forms of child mortality in Bangladesh: application of propensity score matching technique with inverse probability of treatment weighting

Thank you for inviting me to review this manuscript. It is a crystal clear that the problems under the study is nitty-gritty. Hence, the impotency of the topic both locally and globally unquestionable. As the result, I take this opportunity to say thanks for their unreserved effort and dedication.

I do have some comments that I put below.

**Title**

Way used the phrase different forms of child mortality. Its seems vague to understand. Do you think that the child mortality entails neonatal infant under-five death? Hence you are kindly expected came-up with a clear and succinct title.

**Abstract**

Please kindly replace the sub-title of **objective** under the section of the **abstract** with background.

**Methods**

You have to clearly write the study period including the specific date and months. When you are declared statically significance, what was you your sampling procedure? Please omits the way you dichotomize variables such as yes and no from tis sections.

**Result**

What was this numbers indicate 48.8, 30.8, and 23.1. Its numerical numbers or present. Only put your finding rather than comparing with their counterparts as its not recommended to do that

**Conclusion**

Add S in conclusions. Paraphrases **The findings indicate** to This finding indicate that

**Introduction**

Generally your introduction part were well-articulated. But it’s as a few typological, editorial and grammatical such as in paragraph one line 13

R**eparse However, in 2022, when we are at around the middle time of the SDGs’ period, 17 and 37 neonatal and under-five deaths are recorded per 1000 live births, respectively [5]. Importantly, the rate of reduction for the first half of the SDG’s period, from 2015-2021, was not adequate to achieve the relevant SDGs’ targets by 2030 and is even lower than the rates of reduction for the equal number of years in the MDGs’ period in respect to the achieving the relevant MDGs’ targets [5]. Therefore, this is considered an ongoing public health threat, particularly in sub-Saharan Africa and South Asia, though interventions at the maternal and health facility level can help these regions to prevent almost all of these deaths [3].**

Paragraph two line two deaths troll changed to death toll

You need to mention the specific reasons that prompted you to conduct this study at the end of the introduction section

Generally, the introduction sections need an edition for English language and grammar.

**Methods and Materials**

You have to put your study design clearly, discuss setting such as Bangladesh in detail and study periods should be apparently indicated including the specific date and month

Please merge sample size and sampling procedure in one sub-title.

Where is your study population and illegibility criteria? Please deal them in a separate sub-title

All result related data should be moved to the result section. For instance a response rate of 98.8%

If I’m not mistaken you have used stratified or cluster sampling, Have you used the design effect for that in calculations of the sample size

Put it the ethical approval which is written under the section of methods should be moved to immediately after conclusion

**Results**

Under the table two what is your reference to categories the maternal age at a birth and exposure to mass media

What mean by mothers working status

**Discussion**

Your discussion were written
