# Supplementary material for: Effects of short birth interval on different forms of child mortality in Bangladesh: Application of propensity score matching technique with inverse probability of treatment weighting
Source: PLoS One. 2023 Apr 21;18(4):e0284776. doi: 10.1371/journal.pone.0284776 (PMC10121045; doi:10.1371/journal.pone.0284776)
Supplement: Attachment — Submitted filename: Response to reviewers.docx [file pone.0284776.s004.docx]

**Reviewer’s comments and author’s responses**

| **Comments to the author** | **Author’s responses** |
| --- | --- |
| **Reviewer #1** |  |
| The manuscript is corrected and reviewed by me three times in three different times and i think now the manuscript is well prepared and organized | Thank you for your thorough review and revisions of the manuscript. Your input has been valuable in ensuring the manuscript is well prepared and organized. |
| **Reviewer #2** |  |
| Thank you for inviting me to review this manuscript. It is a crystal clear that the problems under the study is nitty-gritty. Hence, the impotency of the topic both locally and globally unquestionable. As the result, I take this opportunity to say thanks for their unreserved effort and dedication.  I do have some comments that I put below. | We thank you for your time to review this manuscript. We have addressed your comments and added a point by point responses below. |
| **Title**  Way used the phrase different forms of child mortality. Its seems vague to understand. Do you think that the child mortality entails neonatal infant under-five death? Hence you are kindly expected came-up with a clear and succinct title. | We examined three distinct types of child mortality, including neonatal mortality, infant mortality, and under-five mortality. Due to concerns about overcrowding the title, we opted to use the term 'various forms of child mortality' instead. We are confident that this accurately conveys the outcomes of our study to readers, and therefore, we have chosen not to make any changes to the title. |
| **Abstract**  Please kindly replace the sub-title of **objective** under the section of the **abstract** with background. | We have added objective sub-title in the revised version of the manuscript. |
| **Methods**  You have to clearly write the study period including the specific date and months. When you are declared statically significance, what was you your sampling procedure? Please omits the way you dichotomize variables such as yes and no from tis sections. | We have clearly mentioned the time when the survey analysed in this study was conducted. Further addition will make this the methods section of the abstract is very busy. We therefore did not make any change in this section. However, we have addressed your concern in the methods section of the main paper. |
| **Result**  What was this numbers indicate 48.8, 30.8, and 23.1. Its numerical numbers or present. Only put your finding rather than comparing with their counterparts as its not recommended to do that | These number represent the prevalence of neonatal, infant and under-five mortality per 1000 live birth. It is clearly presented in the results section. I have copy passed this sentence below-  The prevalence rate of neonatal, infant and under-five mortality was 48.8, 30.8, and 23.1 per 1000 live births, respectively.  No further change is required. |
| **Conclusion**  Add S in conclusions. Paraphrases **The findings indicate** to This finding indicate that | We have added “s” to the word “conclusion”. Moreover, we have revised your referred sentence as follow:  *Findings of this study indicate………..* |
| **Introduction**  Generally your introduction part were well-articulated. But it’s as a few typological, editorial and grammatical such as in paragraph one line 13  R**eparse However, in 2022, when we are at around the middle time of the SDGs’ period, 17 and 37 neonatal and under-five deaths are recorded per 1000 live births, respectively [5]. Importantly, the rate of reduction for the first half of the SDG’s period, from 2015-2021, was not adequate to achieve the relevant SDGs’ targets by 2030 and is even lower than the rates of reduction for the equal number of years in the MDGs’ period in respect to the achieving the relevant MDGs’ targets [5]. Therefore, this is considered an ongoing public health threat, particularly in sub-Saharan Africa and South Asia, though interventions at the maternal and health facility level can help these regions to prevent almost all of these deaths [3].**  Paragraph two line two deaths troll changed to death toll  You need to mention the specific reasons that prompted you to conduct this study at the end of the introduction section.  Generally, the introduction sections need an edition for English language and grammar. | We thank you’re your time to review our manuscript thoroughly.  We have addressed your changes in the revised version of the manuscript.  This issue has already been addressed.  We have provided broad explanation about why this study is conducted. We believe no further addition is required.  We have revised the manuscript to improve it clarity and correct grammatical error. |
| **Methods and Materials**  You have to put your study design clearly, discuss setting such as Bangladesh in detail and study periods should be apparently indicated including the specific date and month  Please merge sample size and sampling procedure in one sub-title.  Where is your study population and illegibility criteria? Please deal them in a separate sub-title  All result related data should be moved to the result section. For instance a response rate of 98.8%  If I’m not mistaken you have used stratified or cluster sampling, Have you used the design effect for that in calculations of the sample size  Put it the ethical approval which is written under the section of methods should be moved to immediately after conclusion | We have already provide study design broadly in the “study design and sampling” section of the manuscript. Please have a look.  We believe merging them together make this study harder to understand by the readers. We therefore keep them separate, as it was, in the revised version. Eligibility criteria already presented broadly under the study sample section.  This is not study results, it is sampling process of the survey. It is therefore not logical to write in the results section.  We want to take the opportunity to remain the reviewer that this study is based on analysis of secondary data. We did not collect data from the field. However, during analysis sample design effect was considered. We clearly mentioned it in the statistical analysis section of the manuscript.  We have moved ethical approval section as suggested. |
| **Results**  Under the table two what is your reference to categories the maternal age at a birth and exposure to mass media  What mean by mothers working status | We used well-known criteria to classify maternal age. These groups represent adolescent, adult and late adult. Since this is widely used and well-known fact, we did not focus on them to classify.  We have changed “mothers working status” to “**Mother’s participation in formal work”** |
| **Discussion**  Your discussion were written | Thanks. |
| **Reviewer ##3, 4** |  |
| The manuscript needs a final check for minor typos, grammar and syntax (marked in track changes below). | We have checked the manuscript thoroughly and grammatical errors and typos are corrected. |
| The work presented meets the requirements for publication. There were some unclear statements such as in the inclusive criteria for women in the survey –ii) passed the most recent night before the survey at the selected households - what does this mean? | Thanks to bring this in our attention. We have replaced the word “passed” to “stayed”. I hope this is now easily understandable. |
| Please include the WHO interval range to classify SBI in the discussion. Do you think this SBI range is more applicable for Bangladesh context? If yes/no, why? | We have added WHO cut off point in the discussion section as suggested. We have already described broadly why WHO SBI range is more applicable for Bangladesh. No further addition is required. |
| The reference list needs to be checked for missing DOI and the correct format. | We have checked the references and corrected it as per the PLOS guideline. PLOS journal does support adding DOI in the reference. |
| **Reviewer #5** |  |
| Thank you for your unreserved deeds and works . It is a crystal clear that the problems under the study is nitty-gritty. Hence, the impotency of the topic both locally and globally unquestionable. As the result, I take this opportunity to say thanks for their unreserved effort and dedication. | We also thank you for your time to review this manuscript. |
| **Reviewer #6** |  |
| It was a pleasure reading your article. Thank you for choosing an interesting and essential topic to research.  The article is well written, and I appreciate the previous reviewers' input, which significantly improved your work.  My only suggestion is to add a sentence to the statistical analysis section explaining why you choose the prevalence ratio over the odds ratio in your study.  Keep up the good work, Mona Abdelrehim | We thank you for your time to read this manuscript and provide valuable comments.  The reason for using multilevel Poisson regression analysis, that produced prevalence ratio, is already presented in the manuscript. In the revised version, we have edited it to make it even more clear. |
| **Reviewer #7** |  |
| Thank you for addressing the points that were highlighted in the previous review. The manuscript has merit. | We thank you for your time to minutely read our paper. |
| Reviewer #8: |  |
| The author has describe the objective of the study clearly, methodology was sound and discussion presented well. good job publishing in this topic. more attention should be done in the region as this also may have significant impact post pandemic | We thank you for your time to review this manuscript. Yes, we agree regional level variation need to be considered broadly. However, this is out of this study focus and post-pandemic data is not yet available. The survey we analysed conducted before the pandemic. Future study will be conducted to address this issue. |
